# Unsupervised phenotyping of the periodontal architecture through high‐dimensional clustering of electronic health records: A multicenter study

**DOI:** 10.1002/jper.70123

**Published:** 2026-04-13

**Authors:** Georgios S. Chatzopoulos, Larry F. Wolff

**Affiliations:** ^1^ Division of Periodontology School of Dentistry University of Minnesota Minneapolis Minnesota USA

**Keywords:** cluster analysis, electronic health records, furcation defects, machine learning, periodontitis, precision medicine, tooth mobility

## Abstract

**Background:**

To identify novel periodontal phenotypes using unsupervised machine learning on a large‐scale, multicenter cohort, specifically characterizing disease patterns based on the “periodontal architecture” of localized structural failures (tooth mobility and molar furcation defects) rather than global severity averages alone.

**Methods:**

This cross‐sectional study analyzed electronic health records from 15,723 adult patients with periodontitis. A high‐dimensional feature vector (*D* = 72) was constructed for each patient, integrating tooth‐specific ordinal grades for mobility and furcation, mean probing depths (PPD), clinical attachment loss (CAL), and systemic health variables. Unsupervised phenotyping was performed using principal component analysis (PCA), t‐SNE visualization, and *K*‐means clustering. Cluster validity was assessed via Silhouette Analysis, and phenotypes were compared using ANOVA and Chi‐square (*X* ^2^) tests.

**Results:**

Four distinct architectural phenotypes were identified: (1) Maintenance/Healthy, representing stability; (2) Anterior–Mobility dominant, defined by high‐grade anterior mobility (52.4% prevalence), and the highest diabetes prevalence (12.2%); (3) Molar‐Furcation Dominant, a male‐dominated group (61%) characterized by advanced posterior furcation defects (93.8% prevalence) despite lower anterior mobility; and (4) Generalized severe, exhibiting global architectural collapse. The phenotypes demonstrated statistically significant separation across all clinical metrics (*p* < 0.001).

**Conclusion:**

Periodontitis manifests as distinct architectural archetypes—specifically “Anterior–Mobility” and “Molar‐Furcation” phenotypes—that are often aggregated into a single severity category by traditional staging. These data‐driven clusters have unique systemic risk profiles, suggesting that diagnosis and treatment planning should incorporate the specific localization of structural failure.

**Plain language summary:**

Severe gum disease (periodontitis) is traditionally classified by overall severity, often grouping different types of tooth damage into the same broad category. To uncover hidden patterns, we used an artificial intelligence technique to analyze the detailed dental and medical records of more than 15,000 patients. Instead of simply grouping patients by how advanced their disease was, the computer identified four distinct patient profiles based on specific patterns of tooth damage and overall health. Interestingly, two of the most severe profiles were fundamentally different. One featured loose front teeth and was strongly linked to diabetes and systemic health issues. The other featured damage between the roots of back teeth (molars) and was primarily driven by local anatomical problems rather than general health. These findings demonstrate that severe gum disease is not a single condition, but rather develops through completely distinct biological pathways. Recognizing these unique patterns allows dental professionals to move beyond a “one‐size‐fits‐all” approach, paving the way for personalized treatments—such as medical screening for patients with loose front teeth and targeted surgical repairs for molar damage.

## 1 INTRODUCTION

Periodontitis is a complex, multifactorial inflammatory disease characterized by the progressive destruction of the tooth‐supporting apparatus, mediated by a dysbiotic oral biofilm and a dysregulated host immune response.^1^, ^2^ Traditionally, the diagnosis and management of this condition have relied on clinical phenotypes defined by aggregate severity metrics, such as clinical attachment loss (CAL), radiographic bone loss, and pocket probing depth (PPD).^3^ While these parameters are essential for estimating historical disease experience, they often fail to capture the biological and clinical heterogeneity inherent in the population, potentially obscuring distinct disease subtypes that respond differently to therapy.^4^

The 2017 World Workshop on the Classification of Periodontal and Peri‐Implant Diseases and Conditions introduced a multidimensional staging and grading framework to address these limitations, incorporating complexity and progression risk into the diagnostic lexicon.^3^, ^5^ Although this consensus‐based system represents a significant paradigm shift toward precision medicine, it remains fundamentally expert‐driven, categorizing patients into pre‐determined “boxes” based on linear severity thresholds.^6^ Emerging evidence suggests that periodontal disease does not manifest as a monolithic entity but rather as a spectrum of latent phenotypes driven by unique combinations of local anatomical factors, systemic comorbidities, and host–response profiles, which may not align perfectly with the current staging and grading matrix.^7^, ^8^

Unsupervised phenotyping, a data‐driven methodology utilizing machine learning algorithms such as clustering and latent class analysis, offers a novel approach to disentangling this phenotypic complexity.^9^ By analyzing high‐dimensional patient data without a priori labels, these algorithms can identify naturally occurring, mutually exclusive subgroups that share similar biological or clinical characteristics.^10^ Previous studies utilizing these techniques have successfully identified “periodontal profile classes” (PPC) that demonstrate superior prognostic accuracy for tooth loss and disease progression compared with traditional classification systems alone.^11^, ^12^ However, the external validity of these models has often been constrained by small sample sizes and homogenous cohorts, limiting their generalizability to broader, more diverse clinical populations.^13^, ^14^

A critical gap in the current literature of data‐driven periodontics is the reliance on global averages—such as full‐mouth mean PPD or CAL—which often mask the specific “architectural” distribution of defects across the dental arch.^15^ The localized pattern of structural failure, particularly the involvement of anatomical landmarks such as molar furcations and anterior tooth stability, provides essential diagnostic information that global means often obscure.^16^ Recent epidemiological studies by our group have highlighted that molar furcation defects and tooth mobility are not merely sequelae of severe disease but distinct clinical entities with unique risk profiles and progression trajectories.^17^, ^18^ A patient with generalized horizontal bone loss represents a fundamentally different clinical phenotype than one with localized high‐grade furcation involvement, yet both might be categorized similarly in broad staging systems.^19^

Furthermore, the integration of systemic health data into these unsupervised models remains inconsistent. Periodontitis is bidirectionally linked with numerous systemic conditions, including diabetes mellitus, cardiovascular disease, and metabolic syndrome.^20^ While the current classification incorporates diabetes and smoking as grade modifiers, these factors are often treated as secondary adjustments rather than primary drivers of the phenotypic definition.^21^ Data‐driven taxonomies that simultaneously process oral architecture and systemic burden have the potential to reveal specific “syndromic” phenotypes—such as diabetes‐associated anterior instability—that are currently unrecognized in standard nomenclatures.^22^

To address these limitations, there is a pressing need to apply advanced unsupervised learning techniques to large‐scale clinical repositories, such as the BigMouth Dental Data Repository, which offers the statistical power to detect rare and complex disease patterns.^23^ By utilizing “Big Data” researchers can move beyond simple severity scales and construct high‐dimensional “patient vectors” that capture the full architectural complexity of the oral environment.^24^ This approach aligns with the broader goals of precision oral health, seeking to define disease subtypes based on objective, data‐driven distributions of structural failure rather than subjective consensus.^7^, ^25^

The novelty of this study lies in its focus on the “Periodontal Architecture”—specifically treating tooth mobility and furcation involvement as primary categorical landmarks rather than secondary descriptors—within a massive multicenter cohort. Unlike previous models that focus primarily on pocket depth, this study utilizes a 72‐dimensional feature space to map the spatial distribution of disease. The primary aim of this study was to utilize unsupervised machine learning to identify and characterize novel periodontal phenotypes within a large population of 15,723 patients, determining if data‐driven clustering could reveal clinically distinct architectural patterns. We hypothesized that periodontal disease does not progress along a single linear trajectory of severity, but rather manifests as distinct architectural phenotypes—distinguishable by the specific localization of structural failure (e.g., posterior furcation vs. anterior mobility) and their unique associations with systemic comorbidities. We further hypothesized that these data‐driven phenotypes would provide a more granular risk stratification than can be achieved by traditional global averages alone, potentially identifying high‐risk subgroups that require targeted, and phenotype‐specific interventions.

## 2 METHODS

### 2.1 Study design and setting

This retrospective, cross‐sectional study was conducted using electronic health records (EHR) from the multicenter BigMouth Dental Data Repository, which integrates data from several dental institutions in the United States including Harvard University, University of Texas Health, University of California San Francisco, University of Colorado, Loma Linda University, University of Buffalo, The University of Iowa, and The University of Minnesota. The study utilized clinical and systemic data recorded between 2011 and 2022. The year 2011 was selected as the start date for data extraction because it corresponds with the widespread standardization and implementation of the axiUm EHR system across all participating institutions, ensuring a high degree of uniformity and reliability in the captured clinical data. This retrospective investigation was determined by the University of Minnesota Institutional Review Board (STUDY00016576) not to fall under the category of human subjects research, as defined by the Department of Health and Human Services and the United States Food and Drug Administration. The reporting of this study follows the Strengthening the Reporting of Observational Studies in Epidemiology (STROBE) guidelines.

### 2.2 Participants and selection criteria

The study population consisted of adult patients (≥18 years) who received a diagnosis of periodontitis using Dental Procedure Codes and Current Procedural Terminology (CPT) and underwent at least one comprehensive periodontal examination. To ensure a standardized cross‐sectional baseline for the clustering algorithm, all clinical and architectural data (e.g., probing depths, CAL, furcation, and mobility) were extracted specifically from each patient's initial comprehensive periodontal evaluation. This approach ensures that the identified phenotypes represent the patient's baseline, untreated disease state at the exact time of diagnosis, prior to the influence of any active periodontal intervention. Inclusion required the presence of at least one multi‐rooted molar (for furcation assessment) and a complete set of recorded clinical parameters. Patients with incomplete periodontal records or those who were pregnant at the time of examination were excluded. The final analytic cohort consisted of 15,723 patients.

### 2.3 Data sources and variable measurement

The primary data for this study were retrieved from the BigMouth Dental Data Repository, a centralized data warehouse managed by the University of Texas Health Science Center at Houston. This repository integrates longitudinal EHR from multiple dental institutions. Expert data analysts at University of Texas Health facilitated the extraction process, ensuring the integrity and anonymization of all patient records. The clinical periodontal metrics utilized in the analysis consisted of PPD, CAL, and bleeding on probing (BOP). These parameters were recorded at six distinct sites for every present tooth, including the distofacial, facial, mesiofacial, distolingual, lingual, and mesiolingual surfaces. For this investigation, these site‐specific measurements were aggregated to determine patient‐level mean values for PPD and CAL. Additionally, the inflammatory burden was quantified by calculating the total number of sites exhibiting BOP across the entire dentition.

Architectural landmarks, which represent the structural integrity of the periodontium, were assessed through the evaluation of tooth mobility and molar furcation involvement. Tooth mobility was determined during clinical examination and staged according to the Miller classification system, which grades mobility on an ordinal scale from 0 to III based on the degree of horizontal and vertical displacement. Similarly, molar furcation defects were identified and graded using the Glickman classification, which categorizes the extent of horizontal bone loss within the furcation area on a scale of 0–IV, ranging from incipient involvement to through‐and‐through defects.

Both mobility and furcation grades were treated as ordinal categorical variables to preserve the hierarchical nature of structural failure. Demographic and systemic variables were extracted from the self‐reported medical history sections of the electronic records. These included patient age, sex, and current smoking status, which was categorized as never, former, or current smoker. Systemic health indicators specifically focused on the presence of diabetes and hypertension. Diabetes mellitus and hypertension were specifically selected for inclusion in the clustering model because they represent the most prevalent and reliably recorded systemic comorbidities within the EHR database that possess an established, direct biological impact on the periodontal microvasculature and host inflammatory response. Furthermore, a “Medication Burden” variable was derived by calculating the total count of unique prescription medications actively being taken by the patient at the time of the periodontal examination, serving as a proxy for overall systemic comorbidity. This was selected acknowledging the inherent limitation that polypharmacy can occasionally misrepresent true medical severity in patients with multiple mild conditions.

### 2.4 Architectural feature engineering (the “patient vector”)

To facilitate the unsupervised phenotyping process, a high‐dimensional feature vector was constructed to represent the “periodontal architecture” of each individual patient. This vector, consisting of 72 distinct dimensions (*D* = 72), served as the numerical input for the machine learning pipeline and was designed to capture a holistic snapshot of both localized structural failure and global disease status. The core of this vector comprised tooth‐specific architectural data, specifically the maximum recorded furcation grade and the maximum mobility grade for each of the 32 possible tooth positions in the human dentition. In cases where a tooth was missing or did not exhibit clinical involvement, the corresponding dimensions were encoded as zero, signifying the absence of architectural failure at that specific anatomical site.

Beyond localized tooth data, the vector integrated global periodontal status indicators to provide context for the architectural landmarks. This included the inclusion of the calculated patient‐level mean PPD and mean CAL, as well as the total count of sites with BOP. By including these continuous metrics, the vector balanced site‐specific structural information with the overall severity of the inflammatory and destructive process. The final components of the feature vector consisted of a comprehensive systemic profile. This included binary indicators for the presence of diabetes and hypertension, the patient's smoking status, and their sex. Continuous variables for age and total medication count were also incorporated to account for the influence of aging and systemic health on the periodontal landscape. This unified 72‐dimensional representation allowed the clustering algorithm to evaluate patients based on the spatial distribution of their disease across the dental arch while simultaneously accounting for their systemic health and global periodontal severity.

### 2.5 Statistical analysis and machine learning pipeline

The statistical analysis and machine learning pipeline for this study followed a multi‐stage framework designed to extract latent architectural patterns from the high‐dimensional periodontal data. Initially, all 72 clinical and systemic variables were subjected to data preprocessing and standardization. Continuous variables such as age, mean pocket depth, and medication counts were scaled to a mean of zero and a unit variance, while the categorical architectural landmarks—specifically the ordinal grades for molar furcation and tooth mobility—were maintained as integer‐encoded vectors to ensure that disparate measurement scales did not disproportionately bias the clustering algorithm.

Given the high dimensionality and inherent multicollinearity of the full‐mouth dental arch data—where the periodontal status of adjacent teeth is often highly correlated—the study utilized principal component analysis (PCA) as a primary dimensionality reduction technique. This procedure transformed the original feature space into a set of orthogonal principal components, effectively condensing the 72‐variable architectural profile into the most significant axes of variance while retaining over 95% of the information. This reduction was critical for mitigating the “curse of dimensionality” and providing a more robust input for the subsequent clustering phase. To visualize the underlying structure of the periodontal landscape, *t*‐distributed Stochastic Neighbor Embedding (t‐SNE) was applied to the principal components. This nonlinear dimensionality reduction technique projected the high‐dimensional patient vectors into a two‐dimensional coordinate system, allowing for the visual inspection of structural densities and the natural grouping of the cohort. The perplexity parameter was set to 30 to balance the attention between local and global aspects of the data, which assisted in identifying the distinct “architectural neighborhoods” that characterize different disease manifestations.

The core phenotyping was achieved through the application of *K*‐means clustering to the principal components. This partitioning algorithm was executed iteratively to assign each of the 15,723 patients to one of *K* mutually exclusive clusters based on their proximity to the cluster centroids in the architectural feature space. The optimal number of clusters was determined using a dual‐validation approach: the Elbow method, which identifies the point where increasing *K* yields diminishing returns in reducing the within‐cluster sum of squares (inertia), and the calculation of the Silhouette Coefficient to measure the cohesion and separation of the resulting phenotypes. A Silhouette Score of approximately 0.21 was achieved, confirming the presence of a nonrandom, clinically relevant structure within the BigMouth repository data.

Data normality was assessed using the Kolmogorov–Smirnov test. Due to the large sample size, parametric assumptions were largely met; however, to ensure rigor for multiple group comparisons, robust one‐way Analysis of Variance (ANOVA) was utilized, and all *p*‐values for post hoc pairwise comparisons were adjusted using the Bonferroni correction. Finally, the discriminative power of the discovered phenotypes was validated through comprehensive post‐hoc statistical testing. One‐way ANOVA was performed to compare continuous clinical metrics, such as mean PPD, CAL, and BOP, across the identified clusters. For categorical systemic variables and demographic factors, including diabetes prevalence, smoking status, and sex distribution, Chi‐square (*X* ^2^) tests were employed. The magnitude of separation between phenotypes was quantified using *F*‐statistics, ensuring that the clusters were not only mathematically distinct but also clinically polarized. All statistical significance established at an alpha level of 0.05.

## 3 RESULTS

### 3.1 Cohort and architectural feature mapping

A high‐dimensional analysis was performed on a cohort of 15,723 patients, utilizing a 72‐feature vector to map the “Periodontal Architecture”. This vector integrated continuous clinical parameters (PPD, CAL, and BOP) with categorical, tooth‐specific structural failures: mobility and furcation involvement (graded 0–III). By treating structural loss as categorical architectural landmarks rather than simple averages, we captured the localized patterns of oral destruction often obscured by global indices.

### 3.2 Unsupervised discovery of periodontal phenotypes

**FIGURE 1:**
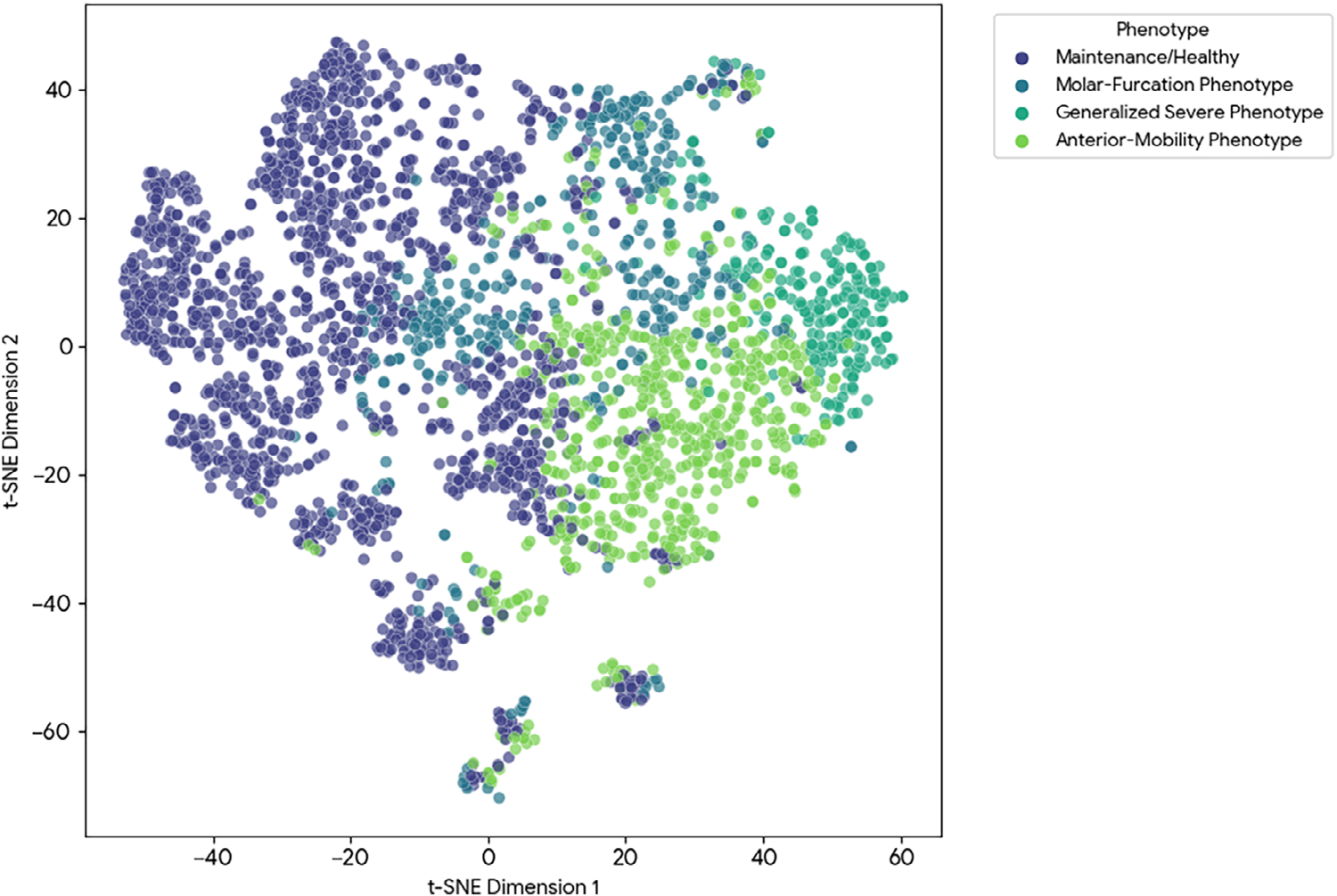
t‐SNE map of periodontal architecture phenotypes. A two‐dimensional visualization of the 72‐feature patient vectors, showing the clear clustering of patients into four distinct architectural archetypes.

**FIGURE 2:**
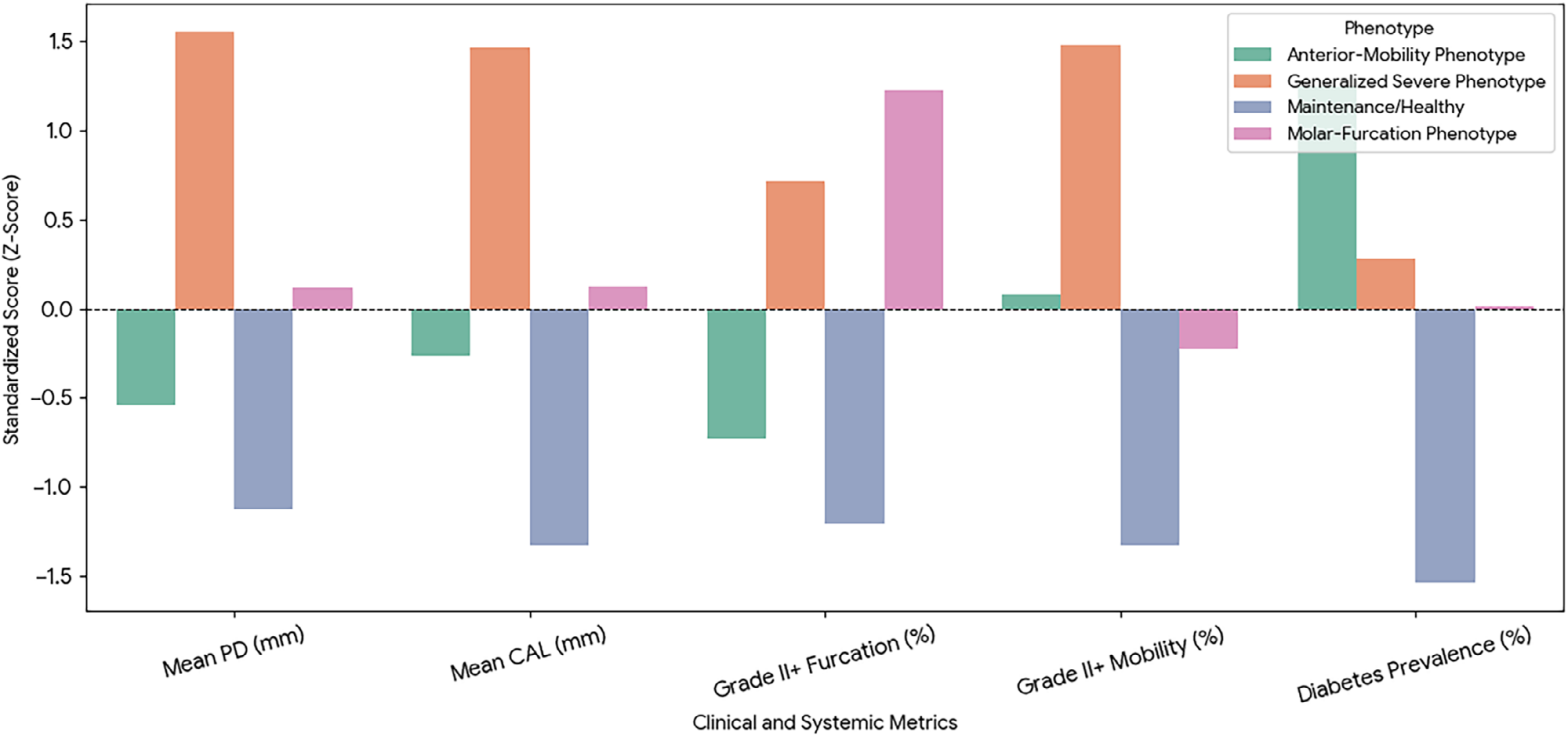
Phenotype profile comparison (standardized scores). Z‐score profiles for each cluster across key metrics. The distinct spikes in mobility for the Anterior–Mobility cluster and furcation for the Molar‐Furcation cluster highlight their unique architectural identities.

The optimal number of clusters was mathematically determined to be exactly *K* = 4 through rigorous evaluation using both the Elbow method (assessing within‐cluster sum of squares or inertia) and the Silhouette Score across a tested range of *K* = 2–10. Specifically, the analysis revealed a distinct inflection point (“elbow”) at *K* = 4 in the inertia curve, which coincided with a maximized Silhouette Coefficient, thereby confirming that four clusters provided the optimal mathematical balance of intra‐cluster cohesion and inter‐cluster separation. Therefore, unsupervised machine learning via t‐SNE and *K*‐means clustering revealed four distinct periodontal archetypes. The spatial separation of these phenotypes in the t‐SNE map (Figure 1) demonstrates that periodontal disease manifests in unique architectural “neighborhoods” rather than a single linear progression of severity. While traditional staging focuses on attachment loss, these clusters are distinguished by the location and type of structural collapse (Figure 2). The detailed demographic, clinical, and systemic characteristics of each phenotype are summarized in Table 1.

**FIGURE 3:**
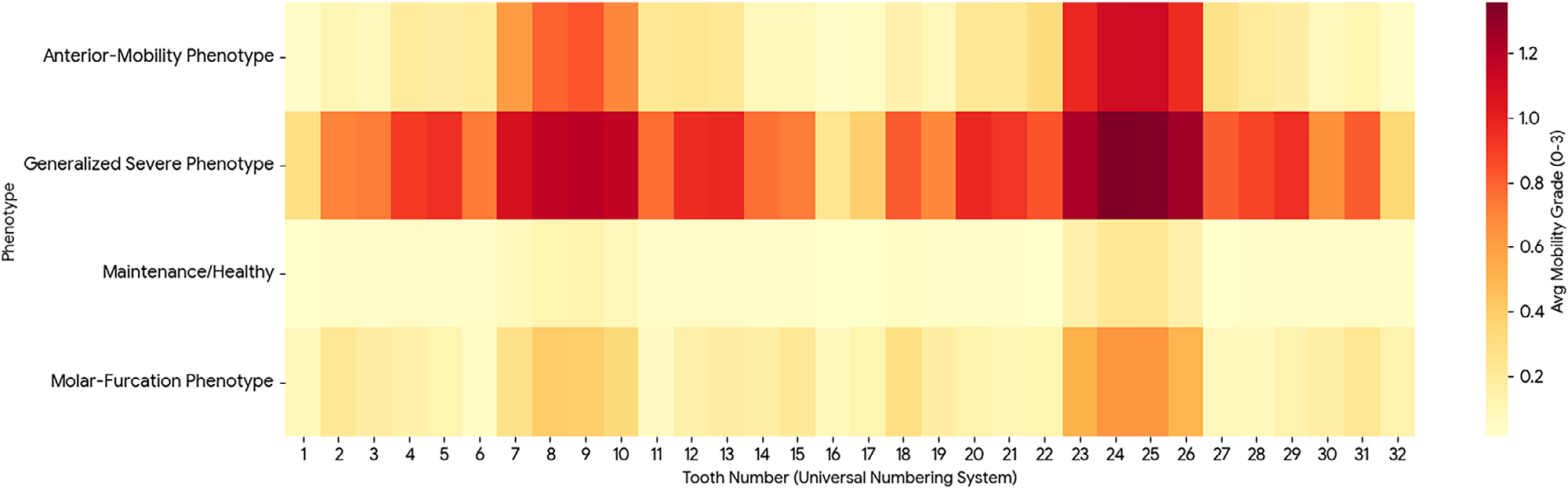
Architectural heatmap of tooth mobility. Heatmap of average mobility grades per tooth. The intensity in the middle of the dental arch for the Anterior–Mobility phenotype demonstrates a localized structural failure.

**FIGURE 4:**
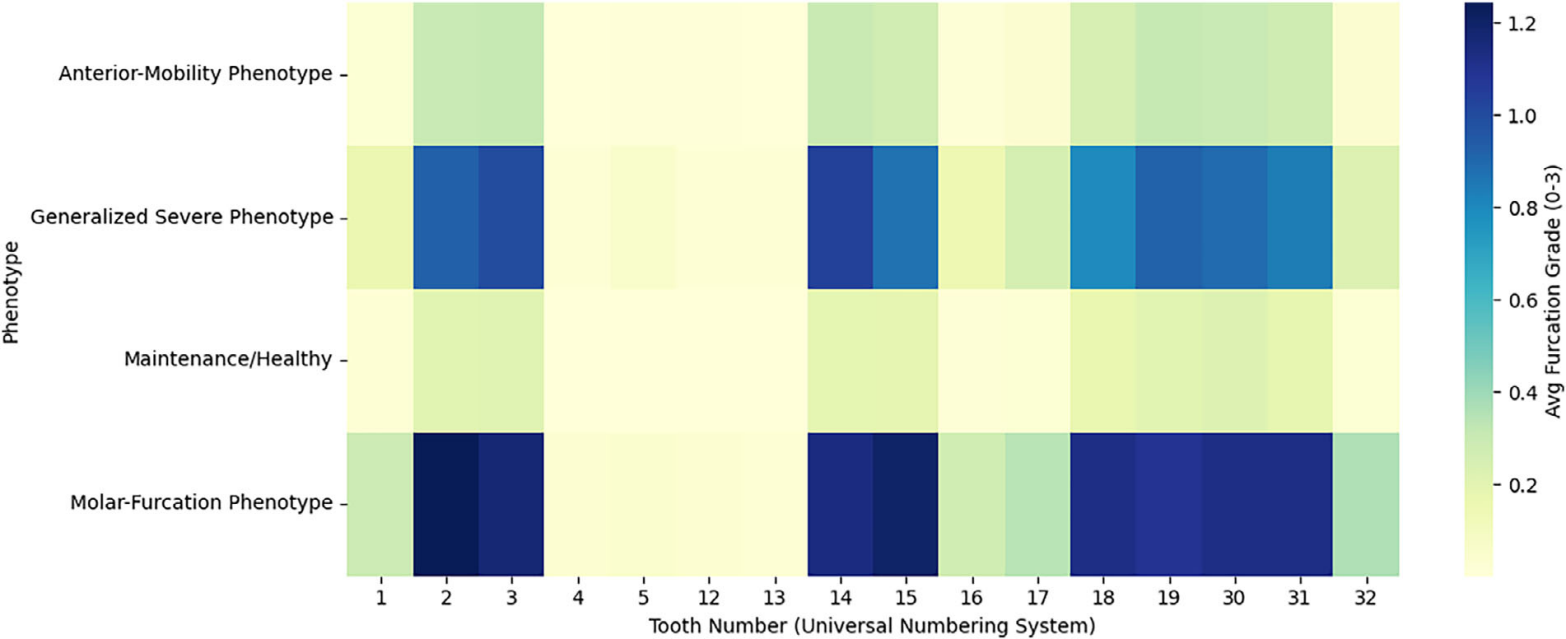
Architectural heatmap of molar furcation. Focused heatmap of molar and premolar teeth, displaying the intensity of furcation grades. This illustrates the specialized posterior destruction characterizing the Molar‐Furcation phenotype.

1. The Maintenance/Healthy Phenotype (*n* = 9283)
  The largest cluster represents architectural stability. Clinical metrics were well maintained (mean PPD: 2.84 mm, CAL: 2.20 mm), and significant structural failures were rare. Only 21.2% of patients exhibited any Grade II+ furcation, and 11.1% showed Grade II+ mobility. This phenotype serves as the architectural baseline for the cohort (Figure 2).
2. The Anterior–Mobility Dominant Phenotype (*n* = 3726)
  This phenotype is defined by a specific structural vulnerability localized to the anterior segments. While global attachment loss was moderate (mean CAL: 2.70 mm), more than half of the patients (52.4%) exhibited high‐grade (Grade II or III) mobility. The architectural heatmap in Figure 3 illustrates this concentration of instability in the mandibular and maxillary anterior teeth. Systemically, this cluster was the most medically burdened, exhibiting the highest prevalence of diabetes (12.2%) and a high medication count (4.26 unique medications).
3. The Molar‐Furcation Dominant Phenotype (*n* = 2237)
  In contrast to the anterior‐centric failure, this cluster manifested a “posterior‐centric” collapse. An overwhelming 93.8% of patients in this group presented with Grade II or III furcation involvement, a structural signature clearly visualized in the molar‐specific architectural heatmap (Figure 4). Despite this severe posterior destruction, anterior mobility remained lower than in the Anterior–Mobility group (43.4%). This phenotype was notably male‐dominated (61%), suggesting a distinct sex‐linked architectural disease pattern.
4. The Generalized Severe/Architectural Collapse Phenotype (*n* = 1344)
  This group exhibited a global failure of the periodontal architecture. It was characterized by the highest mean PPD (3.68 mm), highest CAL (3.51 mm), and extreme scores across both categorical landmarks: 93.7% prevalence of Grade II+ mobility and 78.6% Grade II+ furcation. The combined architectural burden for this phenotype is represented by the extreme standardized scores across all structural metrics in Figure 2.

**TABLE 1:** Categorical and architectural characteristics by phenotype.

Architectural metric	Maintenance	Anterior–Mobility	Molar‐Furcation	Generalized Severe
Grade II+ furcation prevalence	21.2%	35.6%	93.8%	78.6%
Grade II+ mobility prevalence	11.1%	52.4%	43.4%	93.7%
Mean pocket depth (mm)	2.84	03.02	3.23	3.68
Mean attachment loss (mm)	2.20	2.70	2.88	3.51
Diabetes prevalence	7.3%	12.2%	10.0%	10.5%
Sex (male %)	45%	38%	61%	42%

### 3.3 Cluster validation and statistical power

**FIGURE 5:**
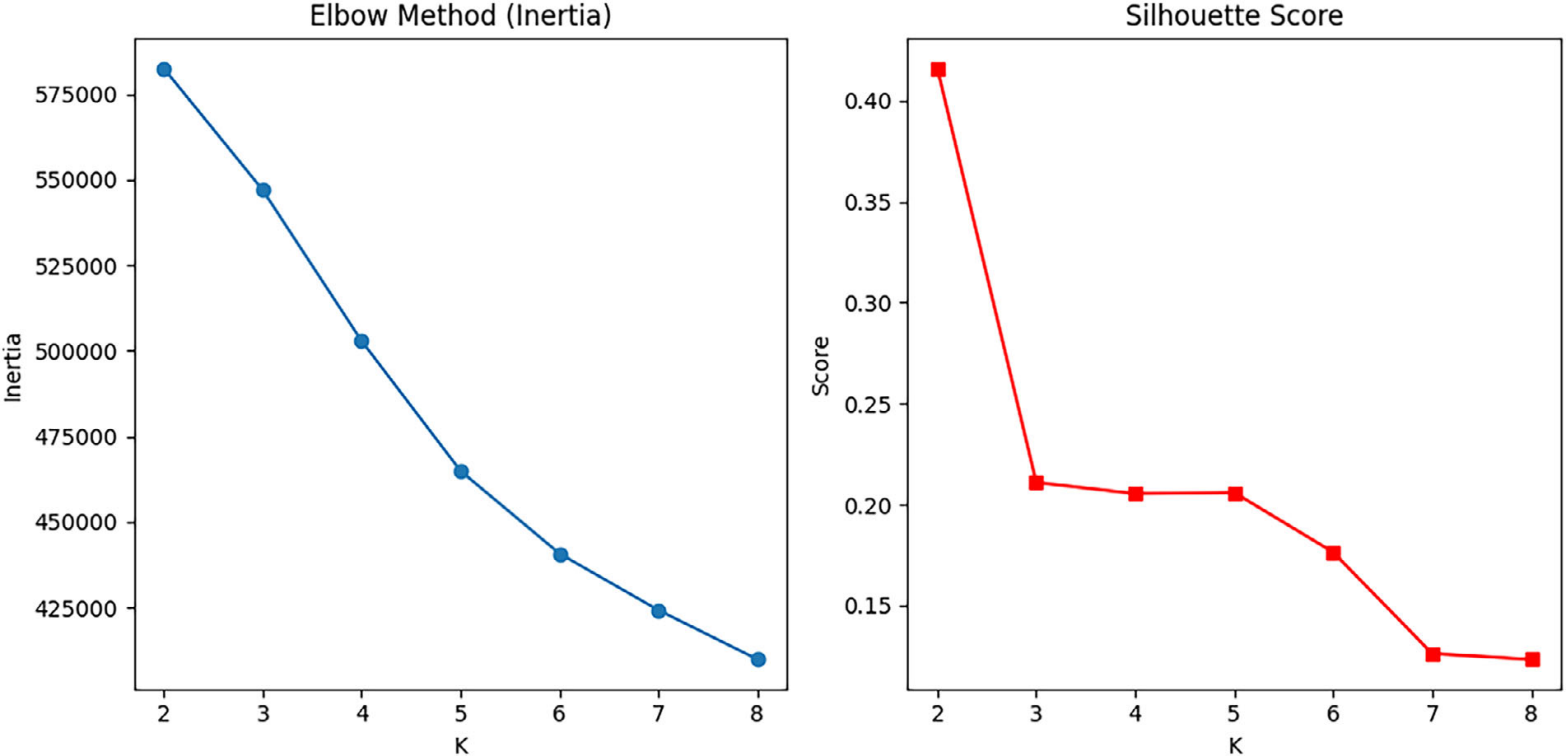
Cluster validity metrics. Elbow method (inertia) and Silhouette Score analysis evaluating the optimal number of clusters (*K*) and the mathematical robustness of the phenotypic separation.

The mathematical validity of the four‐cluster model was assessed using internal consistency metrics (Figure 5). The Elbow method (inertia) confirmed a structural “elbow” between *K* = 4 and *K* = 6, while the Silhouette Coefficient (≅ 0.21) indicated a robust, non‐random clustering structure typical of complex clinical datasets.

The discriminative power of the phenotypes was further validated via one‐way ANOVA across primary clinical indicators. The clusters demonstrated extreme statistical polarization (*p* < 0.0001) for mean PPD (*F* = 787.28), mean CAL (*F* = 1001.21), BOP (*F* = 96.36) and patient age (*F* = 144.58). These high *F*‐statistics confirm that the unsupervised phenotyping effectively partitioned the population into groups with maximum clinical variance, providing high statistical power for the architectural classification.

### 3.4 Comparison of clinical and architectural landmarks

The phenotypes demonstrated highly significant differences in their “architectural fingerprints” (ANOVA, *p* < 0.001). Traditional staging might aggregate the localized failures of the Anterior–Mobility and Molar‐Furcation clusters into a single moderate category; however, our architectural mapping shows they are distinct clinical populations. The divergence between molar‐specific furcation destruction and anterior‐specific mobility suggests that localized structural context provides critical diagnostic information beyond traditional PPD and CAL measurements.

## 4 DISCUSSION

### 4.1 Summary of key findings

The primary outcome of this large‐scale, multicenter study was the identification of four distinct periodontal phenotypes through the unsupervised clustering of 15,723 patient vectors. Confirming our prior hypothesis, the analysis demonstrated that periodontitis does not manifest solely as a linear progression of attachment loss but rather as complex “architectural” patterns defined by the specific localization of structural failure. While the maintenance and generalized severe clusters represented the expected poles of health and disease, the discovery of the Anterior–Mobility Dominant and Molar‐Furcation Dominant phenotypes provides novel insight into the heterogeneity of periodontal pathogenesis. These findings suggest that molar furcation defects and anterior tooth mobility are not merely secondary sequelae of generalized periodontitis but may represent primary architectural landmarks that define distinct disease subtypes with unique systemic and demographic risk profiles.

A notable observation in our phenotypic profiling was that the “generalized severe” phenotype, despite representing the most advanced and destructive disease state, presented with seemingly moderate mean PPD and CAL values. This apparent discrepancy highlights a fundamental limitation of utilizing traditional full‐mouth, patient‐level averages. By aggregating data across the entire dentition, the mathematical impact of localized, extreme deep pockets is inherently diluted by the presence of remaining shallower, healthier sites. This finding directly reinforces the core premise of our study: that relying solely on global severity averages can obscure the true complexity and localized severity of periodontal breakdown, thereby necessitating the more granular, architectural approach provided by unsupervised machine learning.

While these four distinct phenotypes may appear clinically defined by a few dominant features post hoc—such as isolated anterior mobility or posterior furcation involvement—it is critical to emphasize the unsupervised nature of this discovery. The machine learning model was not pre‐programmed or instructed to separate patients based on mobility or furcation grades. Rather, the algorithm arrived at these specific clusters by simultaneously processing a comprehensive 72‐dimensional data vector for every patient. Out of all available clinical, architectural, and systemic variables, the algorithm mathematically discovered that the spatial distribution of these specific structural failures was the primary driver of phenotypic variance across the population.

### 4.2 Interpretation in the context of current classifications

The current 2017 World Workshop classification system represented a significant advancement by introducing a multidimensional staging and grading framework.^3^, ^5^ It elegantly incorporates distinct structural failures—such as advanced tooth mobility (≥ Class II) and severe furcation involvement (Grade II/III)—as essential complexity factors used to define stage III and stage IV periodontitis. However, this expert‐driven consensus relies heavily on global severity metrics. Consequently, patients exhibiting either of these isolated features might simply be diagnosed with “localized severe periodontitis” and universally upgraded into the same broad stage III or stage IV prognostic “box”.^3^ Our findings suggest that traditional staging may inadvertently obscure critical phenotypic differences by collapsing these localized architectural failures under a single umbrella.

By integrating categorical variables for furcation and mobility as primary drivers of our high‐dimensional clustering algorithm rather than secondary complexity factors, we unveiled a level of granularity that significantly supplements the existing matrix.^6^ The primary novelty of our unsupervised clustering approach lies in demonstrating that these two complexities are not simply interchangeable manifestations of advanced localized disease. Rather, our model reveals that “Anterior–Mobility” and “Molar‐Furcation” represent highly divergent, mutually exclusive disease pathways with distinct etiologies.

The clinical significance of this distinction is profound. The Anterior–Mobility phenotype is characterized by systemic vulnerability and anterior instability, demonstrating the highest association with systemic comorbidities (strongly diabetes‐dominated). Clinically, the presentation of this phenotype should serve as a diagnostic “red flag,” prompting immediate systemic and metabolic evaluation, as the rapid destabilization of single‐rooted anterior teeth may predominantly reflect microvascular impairment and a compromised host response. Conversely, the Molar‐Furcation phenotype is primarily driven by posterior anatomy and localized biomechanical vulnerability (notably male‐dominated in our cohort) rather than a systemic burden. For this phenotype, clinical management and tooth survival strategies must heavily prioritize localized regenerative therapies, resective procedures, or early strategic extractions to prevent posterior occlusal collapse.

Ultimately, by disentangling these distinct biological trajectories from the broad “stage III/IV” categories, these machine learning‐driven phenotypes highlight the added value of unsupervised clustering in uncovering the diverse drivers of periodontal breakdown. They move beyond broad clinical descriptors to align with recent calls for “precision oral health”,^4^, ^7^ offering a more granular, biologically aligned roadmap for personalized periodontal intervention.

### 4.3 Comparison with previous unsupervised models

Our results expand upon previous efforts to establish a data‐driven taxonomy for periodontal disease. Earlier studies utilizing latent class analysis, such as the “Periodontal Profile Classes” (PPC), successfully stratified patients based on tooth loss probabilities and extent of disease.^9^, ^11^ However, these models were often limited by their reliance on averaged clinical parameters or homogenous cohorts.^10^ The current study distinguishes itself by utilizing a high‐dimensional feature vector (*D* = 72) that captures the specific spatial distribution of defects. Unlike previous models that might classify a patient simply as “severe”, our model distinguishes where the severity is located. This supports the observation that distinct phenotypes exist based on specific clinical presentations.^8^ Furthermore, recent validation of machine learning models has shown that incorporating granular clinical features significantly improves prognostic accuracy compared with standard definitions alone.^12^

### 4.4 Phenotypic mechanisms and systemic associations

The biological plausibility of these phenotypes is supported by their distinct systemic associations. The Anterior–Mobility dominant phenotype showed the strongest correlation with diabetes mellitus (12.2%). This finding corroborates established evidence linking hyperglycemia to exaggerated inflammatory responses and tissue destruction.^20^ Recent exploratory studies by our group on tooth mobility in this same cohort confirmed that mobility is significantly associated with hypertension and diabetes, acting as a distinct marker of systemic compromise.^18^

Conversely, the Molar‐Furcation dominant phenotype was significantly male‐dominated (61%) and characterized by posterior destruction. This aligns with our group's recent epidemiological findings indicating that furcation involvement is a distinct entity influenced by root anatomy and sex‐specific risk factors.^17^ Furthermore, our longitudinal analysis of furcation progression demonstrated that these defects follow a specific trajectory often independent of global hygiene scores, suggesting that local anatomical factors drive this specific phenotype.^19^ These associations imply the existence of “syndromic” phenotypes, where systemic comorbidities drive specific architectural failures, a concept supported by recent historical reviews on the biological definition of periodontal diseases.^2^

### 4.5 Strengths and limitations

A major strength of this study is the use of the BigMouth Dental Data Repository, providing a massive, multicenter sample (*n* = 15,723) that enhances statistical power and external validity compared with single‐center studies.^23^ The use of unsupervised machine learning minimizes human bias, allowing the data structure to dictate the classification. However, limitations inherent to retrospective EHR analyses must be acknowledged. The data are subject to recording bias, where clinicians may record probing depths or furcation grades more meticulously in diseased sites than in healthy ones.^13^ Additionally, the cross‐sectional nature of the clustering precludes definitive conclusions regarding the temporal progression of these phenotypes. While we observed associations with smoking and medications, the lack of granular biological data (e.g., lipid metabolism markers or immune microenvironment data) limits our ability to define the molecular underpinnings of these architectural patterns.^22^ Furthermore, utilizing medication count as a proxy for systemic health may occasionally misrepresent medical severity, as polypharmacy can occur with mild comorbidities. While total medication count is frequently utilized in large‐scale electronic health record studies as a practical proxy for systemic comorbidity and patient frailty, it is acknowledged that this metric has inherent limitations. Specifically, a higher number of medications does not always perfectly correlate with medical severity, as multiple medications can occasionally be prescribed for the management of several mild conditions.

### 4.6 Implications for clinical care and policy

While the clinical and biological implications of these machine learning‐driven phenotypes require further longitudinal validation, our findings generate several hypotheses regarding the future of periodontal management. The identification of distinct architectural archetypes suggests that traditional severity‐based classifications might eventually be supplemented by phenotype‐specific care pathways. For instance, if the strong association between the Anterior–Mobility phenotype and systemic comorbidities is prospectively confirmed, it could potentially inform more targeted medical‐dental screening protocols. From a health policy perspective, recognizing these distinct trajectories may eventually help to refine resource allocation–perhaps suggesting a need for specialized interventions –likely involving resective or regenerative molar therapy–for Molar‐Furcation patients and enhanced systemic management and splinting for Anterior–Mobility patients.^15^ Ultimately, these findings should be viewed as a foundational, hypothesis‐generating step toward precision periodontics, necessitating future prospective trials before any formal integration into routine clinical guidelines or policy frameworks.

Recognizing these distinct phenotypes could lead to more targeted care pathways, moving away from a “one‐size‐fits‐all” approach, as highlighted in recent consensus reports on emerging diagnostic technologies.^21^ Furthermore, the application of such data‐driven frameworks to epidemiological surveys could refine our understanding of disease prevalence, as recently demonstrated with the application of the 2018 classification to US survey data.^14^

### 4.7 Future research directions

Future research should focus on the longitudinal validation of these phenotypes to determine if they predict tooth loss or responsiveness to therapy differently. Recent predictive models developed by our group have shown that distinguishing between stable and progressive disease requires large‐scale longitudinal data.^24^ Specifically, prospective studies should investigate whether the Anterior–Mobility phenotype represents a rapid‐progression group that benefits from systemic host modulation. Additionally, machine learning models predicting 10‐year tooth loss have emphasized the importance of molar‐specific parameters,^16^ and future work should integrate these architectural clusters to improve prognostic precision.^25^

## 5 CONCLUSIONS

In conclusion, this study demonstrates that periodontal disease is characterized by distinct architectural phenotypes that transcend simple linear severity scales. By leveraging unsupervised machine learning on a large‐scale cohort, we identified unique subgroups defined by the localization of structural failure—specifically molar furcation defects versus anterior mobility—and their differential associations with systemic health. These findings challenge the monolithic view of periodontitis and support a shift toward a precision medicine framework, where diagnosis and treatment are tailored not just to the stage of the disease, but to the specific architectural and systemic profile of the individual patient.

## AUTHOR CONTRIBUTIONS

Both authors have made substantial contributions to the work. **Georgios S. Chatzopoulos**: Contributed to the conception and design of the work and the acquisition of data; analysis and interpretation of data for the work; drafting the manuscript. **Larry F. Wolff**: Contributed to the conception and design of the work and the acquisition of data; revising the work critically for important intellectual content. Both authors give final approval of the version to be published and agree to be accountable for all aspects of the work in ensuring that questions related to the accuracy or integrity of any part of the work are appropriately investigated and resolved.

## CONFLICT OF INTEREST STATEMENT

The authors have no conflicts of interest related to this work.
